# Pediatric spermatic cord fibrolipoma presenting as acute scrotum: a rare case report and literature review

**DOI:** 10.1093/jscr/rjag244

**Published:** 2026-04-09

**Authors:** Francesca Ruspi, Emanuela Lacapra, Ilaria Manghi, Greta Chiastra, Michela Maffi, Simone D’Antonio, Michele Libri, Tommaso Gargano, Marcello Domini

**Affiliations:** Pediatric Surgery Department, IRCCS Azienda Ospedaliero-Universitaria di Bologna, via Massarenti 9, Bologna 40138, Italy; Pediatric Surgery Department, IRCCS Azienda Ospedaliero-Universitaria di Bologna, via Massarenti 9, Bologna 40138, Italy; Pediatric Surgery Department, IRCCS Azienda Ospedaliero-Universitaria di Bologna, via Massarenti 9, Bologna 40138, Italy; Pediatric Surgery Department, IRCCS Azienda Ospedaliero-Universitaria di Bologna, via Massarenti 9, Bologna 40138, Italy; Pediatric Surgery Department, IRCCS Azienda Ospedaliero-Universitaria di Bologna, via Massarenti 9, Bologna 40138, Italy; Pediatric Surgery Department, IRCCS Azienda Ospedaliero-Universitaria di Bologna, via Massarenti 9, Bologna 40138, Italy; Pediatric Surgery Department, IRCCS Azienda Ospedaliero-Universitaria di Bologna, via Massarenti 9, Bologna 40138, Italy; Pediatric Surgery Department, IRCCS Azienda Ospedaliero-Universitaria di Bologna, via Massarenti 9, Bologna 40138, Italy; Pediatric Surgery Department, IRCCS Azienda Ospedaliero-Universitaria di Bologna, via Massarenti 9, Bologna 40138, Italy

**Keywords:** fibrolipoma, paratesticular tumors, acute scrotum, diagnostic laparoscopy, case report, pediatric urology

## Abstract

Fibrolipoma of the spermatic cord is an exceptionally rare benign tumor in the pediatric population that seldom presents as acute scrotum. Only one pediatric case has been reported. We present the second known case, highlighting diagnostic and therapeutic challenges. A 12-year-old boy presented with a 10-day history of right inguinal pain and swelling. Examination revealed a tender inguinal mass with normally positioned painless testes. Ultrasound demonstrated a hyperechoic lesion within the inguinal canal suspected to be an incarcerated omental hernia. Laparoscopy showed obliterated inguinal rings; subsequent open exploration revealed a well-circumscribed fibroadipose mass arising from the spermatic cord. Cord-sparing excision was performed, and histopathology confirmed the diagnosis of fibrolipoma. Outcome was favorable. Fibrolipoma of the spermatic cord should be considered in the differential diagnosis of acute inguinal masses. Laparoscopy can play a pivotal role both for diagnostic clarity and therapeutic benefit. A literature review is performed to contextualize our case.

## Introduction

Acute scrotum represents a common pediatric surgical emergency that requires prompt evaluation to avoid irreversible testicular damage. The most frequent causes include testicular torsion, torsion of the appendix testis and incarcerated inguinal hernia [1]. However, less common etiologies must be considered to ensure an adequate differential diagnosis.

Among the rare causes of inguino-scrotal masses are benign tumors of the spermatic cord, such as lipomas. Fibrolipoma, a benign neoplasm composed of mature adipose and fibrous tissue, is exceedingly uncommon in children. It usually presents as an asymptomatic mass and it is often discovered incidentally during inguinal hernia repair, but in some cases it may mimic acute conditions such as incarcerated inguinal hernia or testicular torsion.

We report the second known case of fibrolipoma of the spermatic cord in a pediatric patient presenting with an acute scrotum. A review of the existing literature is also provided, emphasizing the diagnostic and therapeutic challenges. This report follows CARE guidelines.

## Case presentation

A 12-year-old boy presented to the Emergency Department with a 10-days history of progressive right inguinal pain that had begun following mild physical activity. The pain was described as constant and exacerbated by movement. In the days preceding admission, the patient reported worsening symptoms accompanied by fever. He had no relevant past medical history and appeared in good general condition on presentation. Upon examination a tender swelling was noted in the right inguinal region, involving the entire course of the inguinal canal and markedly painful to palpation. Both testes were located in the scrotum and were normal in volume and consistency with no pain evoked by palpation.

**Figure 1:**
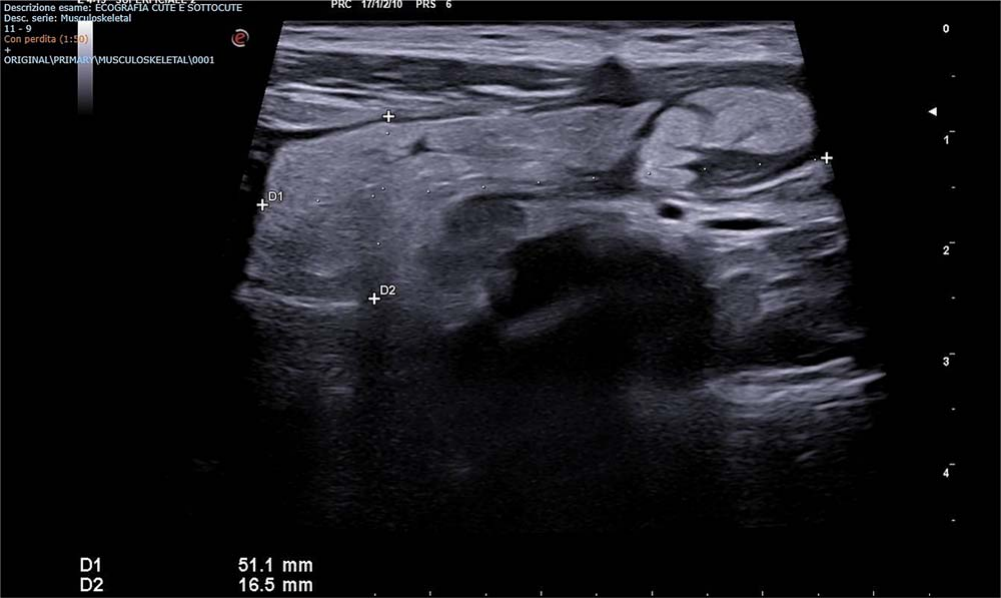
Emergency Department ultrasound images showing a hyperechoic mass occupying the right inguinal canal.

**Figure 2:**
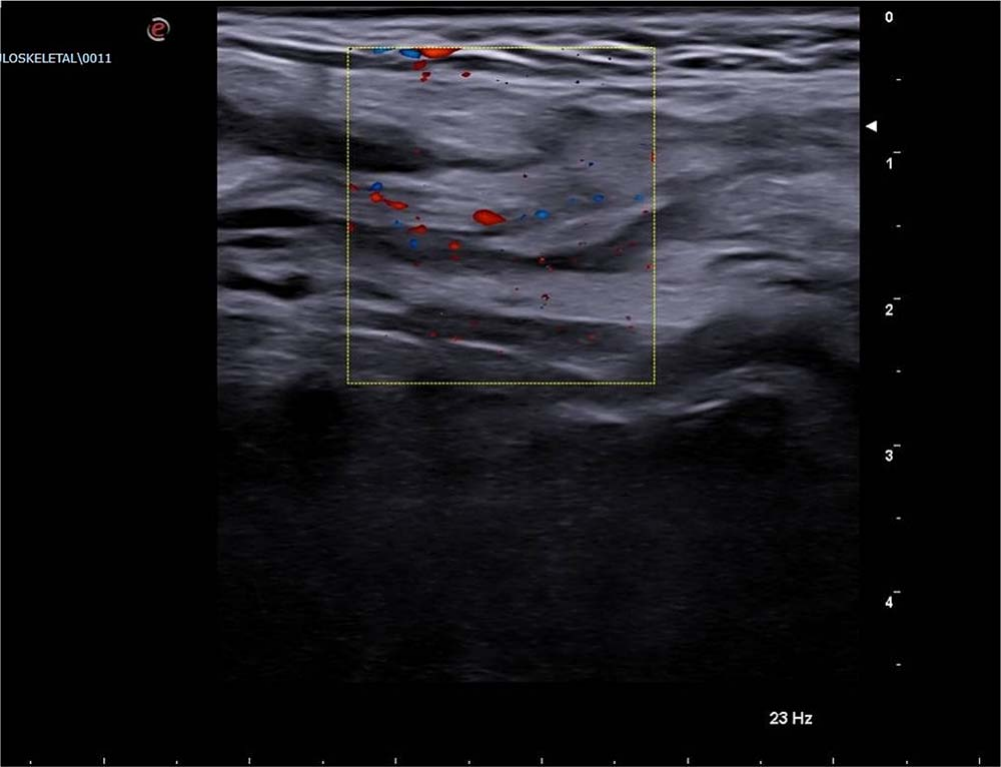
Emergency Department ultrasound images showing a hyperechoic mass occupying the right inguinal canal, with fine vascular signals on color Doppler. The lesion exhibited no change during the Valsalva maneuver and was initially suspected to be an incarcerated omental hernia.

A scrotal ultrasound revealed a grossly hyperechoic area within the right inguinal canal measuring 50 × 20 mm associated with fine vascular spots on Doppler imaging, initially interpreted as thickened omental adipose tissue. No significant changes were observed during Valsalva maneuver, raising suspicion for an incarcerated omental hernia (Figs 1 and 2).

Based on the clinical and imaging findings, surgical exploration was indicated. An exploratory laparoscopy was performed, showing bilaterally obliterated inguinal rings without evidence of herniation. Given these inconclusive findings, open inguinal canal exploration was carried out, revealing a well-circumscribed fibroadipose mass within the canal, of adipose nature with inflammatory features and easily dissectable from the spermatic cord structures. In view of the findings consistent with a spermatic cord lipoma, the lesion was completely excised and sent for histopathological examination.

Collaterally, blood tests including tumor markers (β-HCG and AFP) were performed and resulted negative. The subsequent histopathological examination confirmed the adipose nature of the lesion as a fibrolipoma, showing features of acute and chronic inflammation with areas of steatonecrosis.

Postoperative course was uneventful, with early mobilization and complete resolution of the pain. The patient was discharged on postoperative day one. Outpatient follow-up visits at 1 and 6 months revealed no complications or recurrence.

## Discussion and conclusions

Fibrolipoma of the spermatic cord represents a very rare benign paratesticular neoplasm. Paratesticular tumors account for only 7%–10% of all intrascrotal tumors, and spermatic cord-originating lesions are particularly uncommon [2]. To our knowledge, only one case of fibrolipoma has been reported in the pediatric population [3]. Most paratesticular tumors in children are benign, but the rarity of fibrolipoma contributes to diagnostic uncertainty. In most cases, they present as a mobile and often asymptomatic inguinal mass, which may occasionally be discovered during inguinal hernia repairs [4–6], but they can also present with acute symptoms mimicking more urgent conditions. In the first pediatric case described by Mansiroglu *et al.*, the lesion presented with acute scrotal-like symptoms [3]. Similarly, in the adult literature, spermatic cord lipomas have been reported to present with inguinal pain and bulging, even in the absence of a true hernia sac [6].

In the context of pediatric acute scrotum, the standard differential diagnosis include torsion of the testis, torsion of the appendix testis, epididymo-orchitis or incarcerated hernia. Large retrospective series confirm that testicular torsion and epididymitis account for the majority of pediatric acute scrotum cases [7]. These common etiologies, combined with the rarity of paratesticular tumors in children, often lead to paratesticular masses being overlooked during the initial evaluation [8].

From a diagnostic standpoint, ultrasonography (US) remains the first-line imaging modality, as it is widely available, noninvasive and effective for differentiating intratesticular from extratesticular masses [9]. However, reliable distinction of fibrolipoma from other fat-containing or solid masses on US can be challenging. Moreover, malignancies such as liposarcoma may also present as fatty masses, and their vascular echographic patterns can overlap with benign lesions [10]; for this reason, laboratory tests (e.g. β-HCG and AFP) may be considered in case of malignancy suspect [3]. Köckerling and Schug-Pass noted that clinical diagnosis of spermatic cord lipoma can be unreliable, and imaging with US, CT, or MRI may be necessary [6]. Advances in MRI and particularly sequences with fat suppression could potentially improve diagnostic precision, however, given the rarity of fibrolipoma and the urgency associated with possible acute scrotal presentation, MRI is often bypassed to expedite surgical exploration. Due to the overlap in imaging, definitive diagnosis often relies on histopathology [5].

Given these challenges, surgical exploration becomes a critical component not only for management but also for diagnostic confirmation. In our case, exploratory laparoscopy proved invaluable: it excluded herniation, documented obliterated inguinal rings, and guided the surgeon toward an open inguinal dissection. This underscores how laparoscopic assessment can serve as a minimally invasive yet informative tool in ambiguous acute scrotum presentations.

Once the mass is identified, complete excision with preservation of testicular structures is performed, followed by histological analysis. Moreover, in all reported cases, no recurrence was documented, but long-term follow-up is advised given the rarity of the entity and the limited pediatric experience [6, 10].

In summary, though fibrolipoma of the spermatic cord is rare in children, it should be considered in the differential diagnosis of acute scrotum to avoid delays in management and ensure timely, evidence-based treatment. When clinical and radiological findings are inconclusive, exploratory laparoscopy should be considered as a diagnostic minimally invasive tool, complementing or preceding conventional open surgery, as demonstrated in our case.

## Data Availability

Please contact author for data requests.
